# Physiological and Biochemical Responses in Microalgae *Dunaliella salina*, *Cylindrotheca closterium* and *Phormidium versicolor* NCC466 Exposed to High Salinity and Irradiation

**DOI:** 10.3390/life13020313

**Published:** 2023-01-22

**Authors:** Wassim Guermazi, Salma Masmoudi, Neila Annabi Trabelsi, Sana Gammoudi, Habib Ayadi, Annick Morant-Manceau, George N. Hotos

**Affiliations:** 1 Laboratory of Marine Biodiversity and Environment (LR18ES/30), University of Sfax, Sfax CP 3000, Tunisia; 2 LUNAM, Laboratoire Mer, Molécules, Santé (EA 2160), Université du Maine, Avenue Olivier Messiaen, CEDEX 9, 72085 Le Mans, France; 3 Plankton Culture Laboratory, Department of Fisheries and Aquaculture, University of Patras, 30200 Messolonghi, Greece

**Keywords:** light-salt stress, microalgae, *Dunaliella salina*, *Cylindrotheca closterium*, *Phormidium versicolor*, PSII, photosynthetic activity, antioxidative enzyme activity

## Abstract

*Dunaliella salina* (Chlorophyceae), *Phormidium versicolor* (Cyanophyceae), and *Cylindrotheca closterium* (Bacillariophyceae) were isolated from three ponds in the solar saltern of Sfax (Tunisia). Growth, pigment contents, and photosynthetic and antioxidant enzyme activities were measured under controlled conditions of three light levels (300, 500, and 1000 µmol photons m^−2^ s^−1^) and three NaCl concentrations (40, 80, and 140 g L^−1^). The highest salinity reduced the growth of *D. salina* and *P. versicolor* NCC466 and strongly inhibited that of *C. closterium*. According to ΦPSII values, the photosynthetic apparatus of *P. versicolor* was stimulated by increasing salinity, whereas that of *D. salina* and *C. closterium* was decreased by irradiance rise. The production of carotenoids in *D. salina* and *P. versicolor* was stimulated when salinity and irradiance increased, whereas it decreased in the diatom. Catalase (CAT), Superoxide dismutase (SOD), and Ascorbate peroxidase (APX) activities were only detected when the three species were cultivated under E1000. The antioxidant activity of carotenoids could compensate for the low antioxidant enzyme activity measured in *D. salina*. Salinity and irradiation levels interact with the physiology of three species that have mechanisms of more or less effective stress resistance, hence different resistance to environmental stresses according to the species. Under these stress-controlled conditions, *P. versicolor* and *C. closterium* strains could provide promising sources of extremolyte for several purposes.

## 1. Introduction

Photosynthetic organisms are able to convert solar energy into biochemical compounds necessary for their growth and the development of trophic networks. In estuarine and coastal environments, photosynthetic organisms are often exposed to salt stress combined with light stress [1], particularly in saltworks composed of shallow ponds of increasing salinity [2,3,4]. The combined effects of these two factors have a considerable impact on photosynthetic apparatus [5]. So, organisms inhabiting these paralic ecosystems have developed osmotic adjustment mechanisms to cope with salt stress [6,7,8].

Although primary producers rely on sunlight for photosynthesis, exposure to high levels of active photosynthetic radiation than those required for growth can lead to the inhibition of photosynthesis in algae and plants, particularly during a long period of exposure [9,10]. This photoinhibition affects photochemical reactions by generating reactive oxygen species “ROS” [11], which can oxidize membrane proteins, lipids, and pigments, resulting in membrane instability and photobleaching of the photosynthetic pigments, thus limiting photosynthesis efficiency and growth [12] and threatening the survivability of organisms [5]. To cope with this critical situation, aerobic organisms developed defense mechanisms against ROS accumulation [13] that include antioxidant enzymes (superoxide dismutase, peroxidases, catalase, etc.), non-enzymatic system (carotenoids, ascorbate, glutathione, alpha-tocopherol, etc.) and DNA repair systems [14]. Salt stress can also generate ROS [15]. However, few studies have investigated the induction and regulation of antioxidant defense systems in microalgae under salt stress [16]. 

Under light stress, PSII repair appears to be common and involves the same components in plants and cyanobacteria: proteolytic degradation and synthesis of the new D1 protein, specific phosphorylation (in plants) of several proteins, and PSII migrating damaged complex between the grana regions and stromal thylakoid which is accompanied by changes in the structure of these oligomeric complexes [17]. The repair mechanisms triggered by salt stress are not well clarified in microalgae yet [5]. When in combination with light stress, salt stress enhances the inhibition of PSII in *Chlamydomonas reinhardtii* [18], in leaves of *Hordeum vulgare* and *Sorghum bicolor* [19], and in *Spirulina platensis* [20]. According to Allakhverdiev et al. [5], high light induces photodamage to PSII, whereas salt stress inhibits the photodamaged PSII repair and does not directly accelerate the damage of PSII. The combination of light and salt stress appears to inactivate PSII very rapidly as a consequence of their synergistic effects. The chlorophyll fluorescence technique and photosynthetic oxygen production measured with a Clark-type probe have been regarded as very useful tools for measuring the performance of the photosynthetic apparatus, especially when microorganisms are under stress [20].

Several authors believe that the repair mechanism of PSII in green algae looks like the mechanisms described in land plants, although this aspect is not well studied in algae [21]. In brown algae and diatoms, the mechanisms of photoprotection and repair of PSII have only recently begun to be revealed [22,23]. According to Rohácek et al. [9], microalgae minimize light effects by developing short- and long-term mechanisms to tune the balance between energy utilization and dissipation. Carotenoids play a crucial role in these processes. Indeed, the photosynthetic apparatus is protected against photoinhibition either by thermic dissipation of excess excitation energy in the PSII antenna due to the xanthophylls cycle (non-photochemical quenching) or by transferring electrons from the PSII to different receptors within the chloroplast (photochemical quenching) [24] and finally by dissipating energy as fluorescence [25]. The xanthophyll cycle acts as a photo-protective process that regulates the dissipation of excess light energy [26]. In Chlorophyceae and Phaeophyceae, violaxanthin is de-epoxidized into antheraxanthin and zeaxanthin under excess light [27], and diadinoxanthin is converted into diatoxanthin in diatoms [28].

In order to better understand the physiological and biochemical mechanisms of light and salt tolerance in two microalgae (*Dunaliella salina*, *Cylindrotheca closterium*) and the cyanobacterium *Phormidium versicolor* isolated from an extreme environment like a solar saltern, we investigated in controlled conditions growth rate, photosynthetic pigments, photosynthetic and antioxidative enzyme activities in them. The statistical analysis has allowed us to highlight the most stressful factor for each species.

## 2. Materials and Methods

### 2.1. Isolation and Identification of Microalgae Species

**Figure 1:**
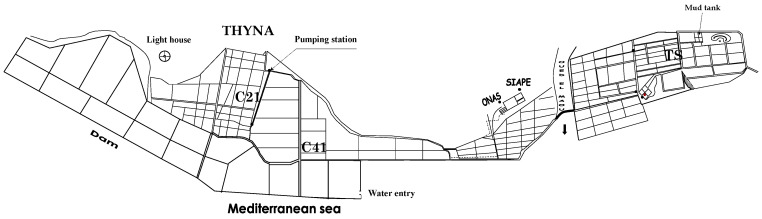
Map of the location with the sampling ponds indicated.

The solar saltern of Sfax (Tunisia, 34° 39′0.1” N and 10° 42′35” E) consists of artificial interconnecting ponds that cover 1500 ha along a 13 km stretch of the Mediterranean coast (Figure 1). Sea salt precipitates under evaporation and is harvested in crystallizing ponds for human consumption. Three autotrophic species: *Dunaliella salina* (Chlorophyceae), *Cylindrotheca closterium* (Bacillariophyceae), and *Phormidium versicolor* NCC466 (Cyanophyceae), were isolated from water samplings collected from TS (mean salinity 346 g L^−1^), C41 (95.5 g L^−1^), and C21 (88.6 g L^−1^) ponds, respectively [2]. Species identification was carried out using morphological criteria, various identification keys, and molecular taxonomy [29,30,31]. 

### 2.2. Culture and Growth Measurement

Monospecific cultures of *D. salina*, *C. closterium*, and *P. versicolor* were carried out into 500 mL artificial seawater [32] under controlled conditions at 24 ± 1 °C and 300 µmol photons m^−2^ s^−1^ provided by white fluorescent tubes (Philips LTD, 18 W) with a light/dark cycle 14 h/10 h. The algal cultures were axenized with antibiotic–antimycotic (10,000 units mL^−1^ penicillin G, 10 mg mL^−1^ streptomycin sulfate, and 25 mg mL^−1^ amphotericin B) treatment (Sigma–Aldrich, St. Quentin Fallavier, France). The initial density of algal cultures was 10^6^ cells mL^−1^ for *D. salina*, 5 × 10^4^ cells mL^−1^ for *C. closterium*, and the initial chlorophyll a (Chl a) concentration of *P. versicolor* cultures was 5 ng mL^−1^. All the experiments were carried out in triplicate. Growth of the eukaryotic algae was measured by cell counting using a Neubauer hemocytometer, while the growth of the filamentous cyanobacterium *P. versicolor* was assessed by determining the Chl a concentration after extraction with dimethyl formamide (DMF) [33] spectrophotometrically. After an acclimation period of 30 days in the conditions previously cited, the three species were grown in artificial seawater (ASW) containing 40, 80, and 140 g L^−1^ of NaCl and exposed to a high irradiance of 300, 500, and 1000 µmol photons m^−2^ s^−1^ (E300, E500 and E1000) provided by white fluorescent tubes for 6, 12, or 13 days to reach the stationary growth phase depending on culture conditions and the species.

Maximum specific growth rate (day^−1^) was determined during the exponential growth phase [34], where x1 and x2 are cell concentrations at t1 and t2 days. The maximum cell density or Chl a content was obtained at the stationary phase.

μ = (Ln x1 − Ln x2)/(t2 − t1)

Photosynthetic pigments in *D. salina* and *C. closterium* were extracted with 90% acetone from 20 mL of algal culture. Photosynthetic pigments in *P. versicolor* were extracted from 10 mL of culture with DMF. Chl a, b, and c were calculated for the Chlorophyceae and the Bacillariophyceae [35] and Chl a for the cyanobacterium [33]. Total carotenoid contents were calculated for *P. versicolor* [36] and *D. salina* [37]. The following equation was used to determine fucoxanthin (Fuco) content in *C. Closterium* [38]:

$$Fuco\;\left(µg\;mL^{-1}\right)=\frac{\left[1000\left(DO473nm-DO750nm\right)-8.08\times Chla\times l-48.64\times Chlc\times l\right]v}{183.4\times l\times V}$$

where v = volume of extract in acetone, V= volume of initial culture, l = optical path (1 cm).

The size of light-harvesting antennas was evaluated by calculating the Chl a/ Chl b ratio in *D. salina* and Chl a/Chl c ratio in *C. Closterium* [27,39].

### 2.3. Molecular Identification

#### 2.3.1. DNA Extraction

The genomic DNA of three species was extracted according to the modified protocol of Doyle and Doyle [40]. Two hundred milliliters of a dense microalga culture was used to extract the genomic DNA. Cells in the exponential growth phase were harvested by centrifugation (4500× *g*, 10 min), and the pellet was washed with distilled water and centrifuged again at 4500× *g* for 10 min. The pellet was then suspended in 3 mL of extraction buffer (0.2% *v*/*v* β-mercaptoethanol, 2% *m*/*v* CTAB, 20 mM EDTA, pH 8, 1.4 M NaCl, and Tris 100 mM, pH 8). After grinding in a mortar pre-fired with liquid nitrogen, the extract was recovered in a 15 mL tube containing 6 mL of extraction buffer previously heated to 60 °C. The tube was then stirred regularly and very carefully for 30 min at 60 °C. After adding 6 mL of chloroform, the mixture was turned over for 5 min, the stopper was opened from time to time to remove the pressure, and then the tube was centrifuged for 15 min at 8000× *g* (4 °C). The aqueous phase was recovered in a new tube, and 4 mL of isopropanol was added to precipitate the nucleic acids. After smooth stirring and incubation for 2 h at −20 ° C, the mixture was centrifuged for 15 min at 8000× *g*, and the pellet was taken up in 500 μL of 70% ethanol. A second wash with 70% ethanol improves the purification of the DNA. After centrifugation, the DNA pellet was dried for 10 min under vacuum or 15 min on the bench and taken up in 80 or 100 μL (depending on DNA dissolution) of TE buffer (1 mM EDTA, pH 8, and Tris-HCl 10 mM, pH 8).

Electrophoresis on agarose gel: The size of the DNA fragment was determined by a 1% (*w*/*v*) of agarose gel electrophoresis by comparing the obtained band with that of the molecular size marker. The gel was prepared in a TAE 0.5×buffer (20 mM Tris-acetate pH8, 0.5 mM EDTA). The migration was carried out in the same buffer for 30 to 60 min at 100 volts. The DNA fragment was then visualized under UV (260 nm) by fluorescence of the ethidium bromide (BEt) in the proportion of 0.1 μg ml^−1^ contained in the DNA.

#### 2.3.2. PCR Amplification and Sequencing

Class-specific and genus-specific primers are generally designed to target the mature ribosomal RNA gene sequences of 16S, 23S, 18S, 5.8S, or 28S, which are considered to be large in size and highly conserved. In addition, internal transcribed sequences (ITS) that are more divergent are also used for species or strain identification [41]. In the present study, Oligonucleotide primers were utilized to amplify the 18S rDNA, 16Sr DNA, 5.8S, and rbcl genes of the tested microalgae (Table 1). Partial genomic DNA sequences of three microalgae species were obtained by the following PCR procedure. PCR was performed in a total reaction volume of 25 μL containing 100 ng of genomic DNA; 0.2 μM of each primer, 0.6 μL of Taq DNA polymerase, 0.5 μL of Taq DNA polymerase buffer, 2.5 mM Mg Cl2 (20 mM), and 0.2 μM dNTP.

The PCR program consisted of 94 °C for 3 min followed by 35 cycles of 94 °C for 30 s, 55 °C for 30 s, and 72°C for 1 min, with an additional 10 min cycle at 72 °C. The PCR product was analyzed by a 1% (*w*/*v*) of agarose gel electrophoresis using a gel/PCR DNA fragment extraction kit (Promega-Wizard SV Gel and PCR Clean up System kit). The purified PCR samples were submitted to Beckman Coulter genomics, England, for sequencing.

The DNA sequences were then aligned using the BLAST web interface (http://blast.ncbi.nlm.nih.gov/Blast.cgi, accessed on 15 March 2021) to the nr (non-redundant) database of NCBI.

### 2.4. Photosynthetic Activity

#### 2.4.1. Oximetry

The rate of net oxygen evolution (PN) of intact cells during exponential growth was monitored with a Clark-type oxygen electrode (Hansatech LTD, UK) under growth conditions as previously described [42]. The oximeter calibration was performed using ASW with NaCl 40, 80, and 140 g L^−1^. The maximum dissolved oxygen concentrations were calculated at 24°C [43]. PN (μmol O_2_ h^−1^ mg^−1^ Chl *a*) was calculated according to the formula

(1) $$P_{N}=\frac{P_{A}\times 60\times 10^{3}}{32\times Chl\;a}$$

where: *P_A_* = Photosynthetic activity (mg O_2_ L^−1^ min^−1^), Chl *a* = mg L^−1^.

#### 2.4.2. Fluorimetry Parameters

Modulated fluorometry is a non-intrusive method providing fast, reliable, and reproducible information on PSII [44]. Chl a fluorescence was measured with 2 mL of algal culture maintained at 24 ± 1 °C with the modified fluorometer FMS-1 (Hansatech Ltd., Cambridge, UK) [45]. The sample was stirred with a magnetic bar placed in the cuvette to ensure the homogeneity of the suspension. Before fluorescence measurement, a period of dark adaptation of samples was applied for 10 min. This period is necessary for a complete re-oxidation of PSII electron acceptors. Then, the measurement of the minimum fluorescence level (F0) and the maximum fluorescence level (Fm) made it possible to calculate the variable fluorescence Fv= Fm − F0 and the maximum quantum efficiency of PSII (Fv/Fm). The steady-state fluorescence (Fs) was measured after 10 min at 300, 500, or 1000 µmol photons m^−2^ s^−1^. A saturating flash induced the maximum fluorescence level of the light-acclimated sample (F’m), and the effective quantum yield efficiency of PSII (ΦPSII) was calculated as: ΦPSII = (F’m − Fs)/F’m. The non-photochemical quenching (NPQ) of fluorescence was determined as: NPQ = (Fm − F’m)/F’m.

#### 2.4.3. Antioxidative Enzyme Activities

Algae and cyanobacteria were harvested by centrifugation (900× *g*, 4 °C) and then immediately frozen in liquid nitrogen. Pellets were transferred into a mortar previously cooled with liquid nitrogen and ground with 1 mL of extraction buffer (sodium phosphate 50 mM, pH 7, EDTA-Na2 1 mM, ascorbic acid 1 mM). The homogenate was centrifuged (10,000× *g*, 15 min, 4 °C), and the supernatant was used for spectrophotometric determination of antioxidative enzyme activities and total protein content. Catalase (CAT) activity was performed [46]. The reaction mixture (phosphate buffer 50 mM, pH 7.5, and 100 µL of extract) was placed in a quartz cuvette at 20 °C. The addition of 100 µL of H_2_O_2_ (200 mM) allowed for the measurement of CAT activity by monitoring H_2_O_2_ reduction at 240 nm for 1 min. The molar extinction coefficient of H_2_O_2_ at 240 nm is 0.04 mM^−1^ cm^−1^. One catalase enzymatic unit corresponds to the quantity of enzyme that degrades 1 µmol H_2_O_2_ per min. Ascorbate peroxidase (APX) activity was assessed for 3 min by the decrease of absorbance at 290 nm due to ascorbate consumption in the presence of H_2_O_2_. The reaction mixture containing phosphate buffer 50 mM, pH 7.5, H_2_O_2_ 0.5 mM, and 100 µL of the microalgal extract was placed in a quartz cuvette at 25 °C. The addition of 50 µL of ascorbate (250 µM) triggered the reaction. A unit of APX is defined as the amount of enzyme needed to consume 1 µmol ascorbate mg^−1^ protein for 1 min. Superoxide dismutase (SOD) activity was determined by measuring the inhibition of photochemical reduction of nitroblue tetrazolium (NBT), which absorbs at 560 nm [47]. The reaction mixture (sodium phosphate buffer 50 mM pH 7.8, NBT 0.57 mM, methionine 5 mM, EDTA 10 mM, Trixton X-100 0.03%, and 100 µL of extract or 100 µL of buffer for the control) was maintained at 25 °C. Riboflavin 10 µM was added to the reaction mixtures that were immediately illuminated with 600 µmol photons m^−2^ s^−1^. Absorbance was measured after 7 min of illumination. One unit of SOD activity was calculated as the enzyme amount required to induce 50% inhibition of the nitro blue tetrazolium (NBT) photoreduction [47]. The protein concentration of each microalgal extract was determined by standardizing with bovine serum albumin [48].

#### 2.4.4. Statistical Analysis

Data are the average ± SE of three independent replicates performed with independent cultures. The data were analyzed by three-way analysis of variance (ANOVA) with two factors: irradiance and NaCl concentration as independent variables. For multiple comparisons, Tukey tests were used. Differences were considered to be significant at a probability *p* < 0.05, 0.01, and 0.001 depending on experience and species. The computational program used was IBM SPSS Statistics version 20.

## 3. Results

### 3.1. Molecular Identification

#### 3.1.1. *Dunaliella salina*

**Figure 2:**
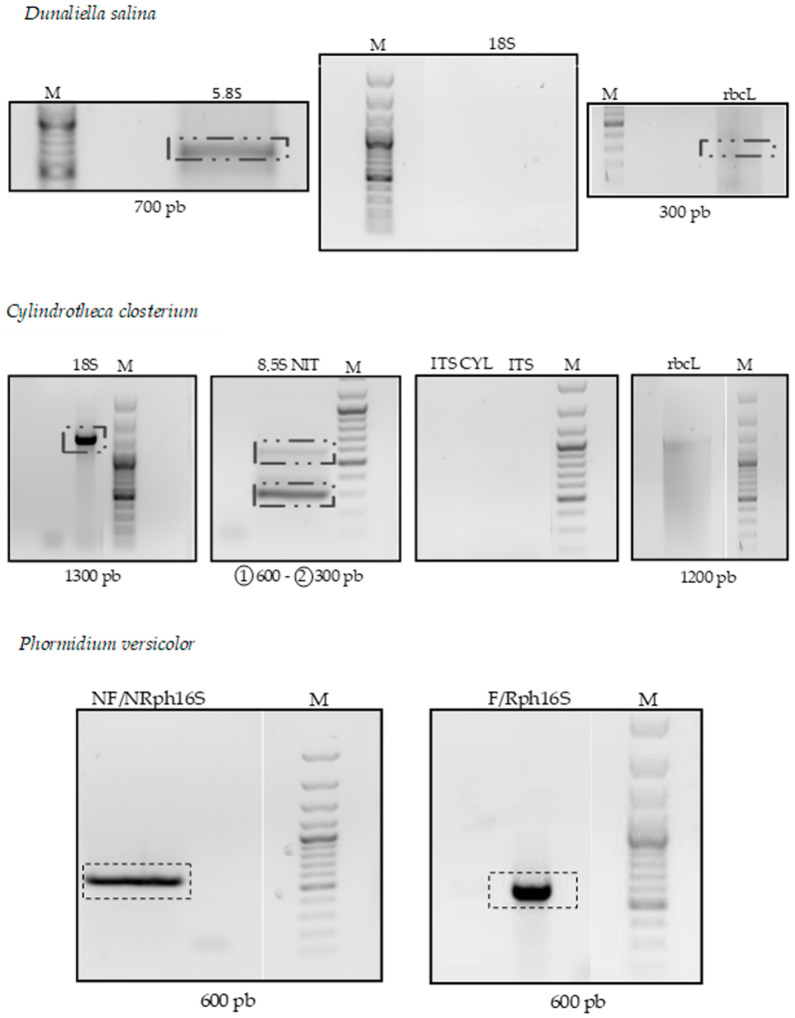
PCR amplification for genes representative of *Dunaliella*: 5.8S, 18S rRNA, and rbcl of *Cylindrotheca closterium*: 5.8S, 18S rRNA, ITS, and rbcl, and of *Phormidium versicolor*: 16S. M: Molecular marker.

Amplifications with 18S primers gave no results (Figure 2). For the ITS gene, PCR amplification with the ITS1 and ITS2 primers gives a 700 bp fragment (Figure 2). The amplified fragment corresponds to the expected size, which varies between 778 and 798 bp for *Pseudo-nitzschia multistriata*. After sequencing, queries in the databases (NCBI) show 100% identity with ITS of *D. salina* (Israelian strain DQ116743.1). Concerning the rbcl gene, the amplified fragment of size 300 bp does not correspond to the expected size (1400 bp). After sequencing and querying databases (NCBI), the sequence corresponds to a nitrate reductase in *D. salina* (AY567972.1).

#### 3.1.2. *Cylindrotheca closterium*

The amplified fragment equal to 1300 bp almost corresponds to the expected size, which is around 1400 bp for the 18S gene (Figure 2). After sequencing and verification by blast from databases (NCBI), the sequence corresponds to 18S of *C. closterium* with 100% similarity. The accession number is DQ178314.1. The alignment shows 100% conservation for this fragment. Concerning the gene 8.5S, the PCR amplification product with the FNitITS2/RNitITS2 primer pair reveals amplifications at the expected size of approximately 300 bp and another of a larger size of 600 bp (Figure 2). After sequencing, whether the fragment has the expected size or not, the two FNitITS2/RNitITS2 primers were found on either side of the fragment. After sequencing and consultation with databases, the fragments do not correspond to the 8.5S gene. Moreover, the alignment of the two fragments (600 and 300 bp) with the ITS2 fragment of Nitzschia sp. shows that there is a similarity only at the level of the primers (Figure 2).

#### 3.1.3. *Phormidium versicolor*

The 16S gene is the gene amplified to determine cyanobacterium. Amplifications of 600 bp, visualized on the agarose gel, correspond approximately to the size expected with NFph16S/NRph16S inner and Fph16S/Rph16S outer primers (Figure 2). After sequencing, the comparison of these amplifications with the sequences recorded in databases proves fruitful. The primers are systematically found from either side of the sequence, and the ClustalW alignment of the sequences shows 100% identity with a 16S sequence of *Phormidium* sp. (AM398796.1). It should be noted that the species is not available in the database, which would allow us to deposit this sequence for the first time with an identification of the cyanobacterium as *P. versicolor* (Accession No. NCC466: *Phormidium versicolor*/Wartmann in Rabenhorst 1861 ex Gomont 1892).

### 3.2. Kinetic Growth under Salt and Light Stress

**Figure 3:**
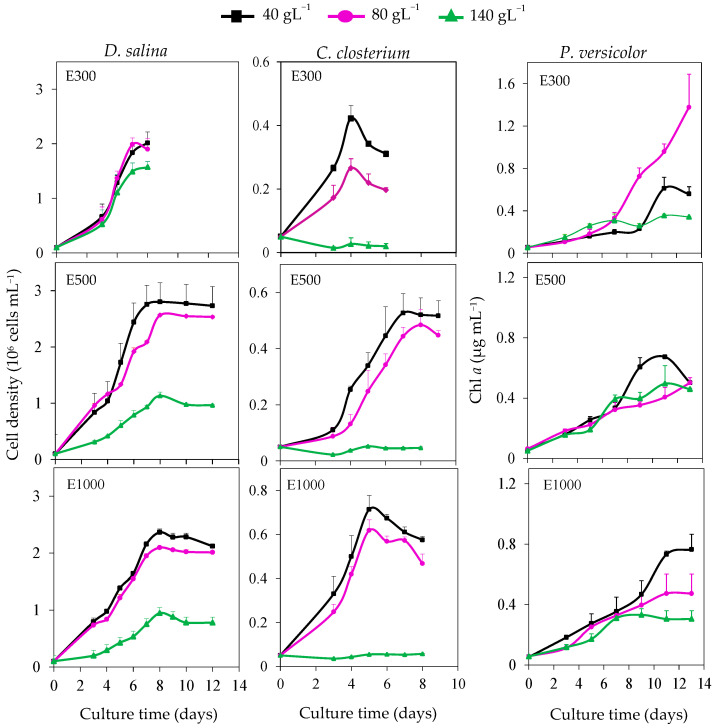
Growth kinetics of *Dunaliella salina*, *Cylindrotheca closterium*, and *Phormidium versicolor* grown in artificial seawater containing NaCl 40, 80, and 140 g L^−1^ under 300, 500, and 1000 µmol m^−2^ s^−1^ (E300, E500, and E1000). Means ± SE (*n* = 3).

In this study, the growth of *D. salina*, *C. closterium*, and *P. versicolor* grown in nine experimental conditions was monitored for 6, 12, or 13 days depending on the light level and the species. An exponential growth pattern was observed at each salt concentration except for *C. closterium* in the presence of NaCl 140 g L^−1^ under E500 and E1000 (Figure 3). Growth curves of *D. salina*, cultivated with NaCl 40 and 80 g L^−1^, were similar whatever the irradiance, and the growth was reduced with NaCl 140 g L^−1^. The Tukey test shows that the different salinities used decreased the maximum cell density (*p* < 0.001) and the maximum growth rate (*p* < 0.01). The maximum cell density increased concomitantly with illumination level from E300 to E500 with NaCl 40 and 80 g L^−1^ (Figure 3, *p* < 0.001, Table 2). The maximum growth rate of *D. salina* was increased when irradiance rose from E500 with NaCl 40 and 80 g L^−1^ (*p* < 0.001). Cell densities obtained with *C. closterium* were lower than those recorded with *D. salina*. The growth of *C. closterium* was slightly higher with 40 than with NaCl 80 g L^−1^ under E300 (Figure 3) and was almost absent at 140 g L^−1^ under the three light levels (Figure 3). The maximum growth rate was null with NaCl 140 g L^−1^ from E500 (Table 2). Maximum cell density decreased with increasing salinity under the three light levels. The maximum cell density of *C. closterium* increased significantly when irradiation reached E1000 with 40 and 80 g NaCl L^−1^ (*p* < 0.001, Table 2). The highest growth of *P. versicolor* was recorded with NaCl 80 g L^−1^ under E300 and the lowest with NaCl 140 g L^−1^ under E1000 (Figure 3).

In *D. salina*, the post hoc test (Tukey) shows a significant decrease of Chl a content with NaCl 140 g L^−1^ under the three light levels (*p* < 0.01) (Table 2). The Chl b content was about three times lower than that of Chl a; the highest concentration was obtained with NaCl 40 g L^−1^ under E300, like that of Chl a. A significant change was observed with NaCl 80 and 140 g L^−1^ under E1000 (*p* < 0.05, Table 2). The light-harvesting antenna size stayed unchanged under the different salt concentrations and light levels (*p* > 0.05). Carotenoid content increased concomitantly with salt concentration (*p* < 0.001). The Tukey test shows a significant increase in these pigments with the highest salinity and the highest irradiance (Table 2). It was not possible to detect photosynthetic pigments in *C. closterium* cells grown in the presence of NaCl 140 g L^−1^ under E500 and E1000 (Table 2). In *C. closterium*, Chl a content decreased significantly (*p* < 0.001) with NaCl 140 g L^−1^ under E300. Chl a concentration decreased significantly (*p* < 0.001) from E300 to E500 with NaCl 40 and 80 g L^−1^ (Table 2) and decreased significantly when NaCl reached 140 g L^−1^ under E300 (*p* < 0.01). Chl c content significantly decreased under higher light levels (E500 and E1000) (*p* < 0.01). Fucoxanthin content followed the same trend as Chl c (Table 2). Chl a/ Chl c ratio increased significantly under E500 and E1000 with NaCl 40 (*p* < 0.001) compared to E300 (Table 2). Chl a content in *P. versicolor* showed a significant decrease with NaCl 140 g L^−1^ under each light level tested (*p* < 0.001, Table 2). Under E300, the accumulation of carotenoids increased when the salinity increased (*p* < 0.001). A higher irradiance led to a significant increase in carotenoids (*p* < 0.001). The maximum carotenoid content was measured with NaCl 80 g L^−1^ under E1000 (Table 2).

### 3.3. Photosynthetic Activity

The net oxygen evolution (PN), on a Chl a basis, was higher in *D. salina* than in *P. versicolor* and *C. closterium* (Table 3). In *D. salina*, PN was almost unchanged up to NaCl 80 g L^−1^ and significantly decreased (*p* < 0.001) with NaCl 140 g L^−1^ under the three light levels. In *C. closterium*, NaCl was the main factor that significantly reduced (*p* < 0.01) PN (Table 3). The addition of NaCl 140 g L^−1^ in the culture medium led to a significant decrease (*p* < 0.001) of PN of *P. versicolor* under the three light levels (Table 3).

In *D. salina*, the maximum quantum yield (Fv/Fm) was equal to about 0.7, whatever the culture condition. The effective quantum yield (ΦPSII) remained the same in the range of 0.3, whatever the salinity under E300; a higher light level (E500 and E1000) induced a significant decreased (*p* < 0.001) of this parameter. Non-photochemical quenching (NPQ) increased concomitantly with the NaCl concentration and the light level (*p* < 0.001, Table 3). A significant increase (*p* < 0.001) of NPQ of about ninefold between E300 and E1000 was observed with the highest NaCl concentration. Due to the absence of growth, fluorescence parameters of *C. closterium* could not be determined in cells cultivated with NaCl 140 g L^−1^ under E500 and E1000. In the other conditions, Fv/Fm was about 0.7, like in *D. salina*. Under E300, a significant decrease of ΦPSII value (*p* < 0.001) of approximately half was observed with NaCl 140 g L^−1^ compared to the lowest salinity (Table 3). ΦPSII did not exceed the value of 0.37 ± 0.02 under E500 and E1000 and was significantly reduced (*p* < 0.001) compared to values obtained under E300. Irradiance and salinity interacted on this parameter as indicated by the Tukey test (F = 0.81, d.f = 9, *p* < 0.001). NPQ increased when NaCl concentration and light level increased (*p* < 0.05, Table 3). In *P. versicolor*, Fv/Fm was lower than values recorded in both microalgae, with an average value of 0.4 (Table 3). As in *D. salina*, no significant variation was observed with the increase in salinity (*p* > 0.05). However, this parameter increased significantly under E1000 with 40 and 80 g L^−1^ compared to E300 and E500. ΦPSII tended to increase with NaCl rising under the three light levels (*p* < 0.001). NPQ of *P. versicolor* changed significantly (*p* < 0.01) with the increase of salinity when this cyanobacterium was grown under E500 (Table 3). E1000 irradiance significantly increased the NPQ of *Phormidium* with NaCl 40 and 80 g L^−1^ (ANOVA, *p* < 0.05).

### 3.4. Antioxidative Enzyme Activities

**Figure 4:**
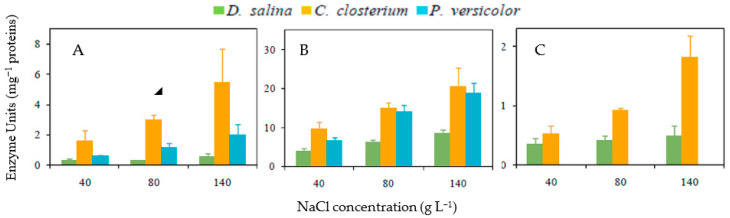
Catalase (**A**), Superoxide dismutase (**B**) and Ascorbate peroxidase (**C**) activities in *Dunaliella salina*, *Cylindrotheca closterium*, and *Phormidium versicolor* grown in artificial seawater containing NaCl 40, 80, and 140 g L^−1^ under 1000 µmol m^−2^ s^−1^. Means ± SE.

Activities of APX, CAT, and SOD were only detected and measured when cells were grown under E1000 (Figure 4). SOD activity was about two times higher in *C. closterium* and *P. versicolor* than in *D. salina*. This enzyme activity increased significantly in *D. salina* (F = 24.68, d.f. = 6, *p* < 0.01), *C. closterium* (F = 8.67; d.f. = 6; *p* < 0.05), and *P. versicolor* (F = 29.78, d.f. = 6, *p* <0.001) when the salinity increased. The highest CAT activity was measured in *C. closterium*, and the lowest was recorded in *D. salina* whatever the salinity. CAT activity increased significantly in each species when the salinity increased (*D. salina*: F = 8.68; d.f. = 6; *p* < 0.05; *C. closterium*: F = 6.20; d.f. = 6; *p* < 0.05, *P. versicolor*: F = 8.21; d.f. = 6; *p* < 0.05). APX activity was not detected in *P. versicolor*. APX activity significantly increased in *C. closterium* when salinity increased (F = 23.76; d.f. = 6; *p* < 0.01), and it stayed almost at the same level in *D. salina*, whatever the NaCl concentration (Figure 4).

## 4. Discussion

This study evaluated the growth and the photosynthetic and antioxidant activities of three halophile microalgae species under light and salinity stress conditions. Their biomolecular signatures have confirmed the determination based on morphological traits we did previously [31]. The growth of the three species studied was differently affected by increasing salinity. The different levels of salt tolerance measured experimentally were in accordance with the distribution of the three species in the solar saltern. Indeed, previous studies [2] have shown that in ponds with a salinity range from 42–96 g L^−1^, Bacillariophyceae, among which *Cylindrotheca closterium*, dominate the other taxa since they represent more than 60% of the total microalgae; Chlorophyceae, represented mainly by *D. salina*, and Cyanophyceae including *P. versicolor*, represented 13 % and 3 %, respectively. In ponds in which salinity ranged from 190–340 g L^−1^, Chlorophyceae and Cyanophyceae were relatively abundant (31% and 70%, respectively) [2]. Our results confirmed that NaCl 140 g L^−1^ decreased at different light levels the growth of *D. salina* and *P. versicolor* and inhibited the growth of *C. closterium*. Moreover, the maximum growth rate in *D. salina* decreased significantly at NaCl 140 g L^−1^ when the light level increased, whereas a significant increase was shown with NaCl 40 and 80 g L^−1^ under E500 and E1000. Salinity and irradiance were the main determining factors in growth rate variation [4,49]. Our results confirm that *D. salina* and *P. versicolor* resist salt stress and that the diatom *C. closterium* is salt sensitive, as it is observed in saltworks. Net photosynthesis values of the three species studied are in accordance with growth curves. Against salinity stress, each species develops different physiological mechanisms that are more or less efficient. *D. salina* is devoid of a rigid polysaccharide cell wall, giving it the ability to adapt to high NaCl concentrations reaching saturation [50]. This adaptability is due to plasma membrane plasticity, which prevents the break and apoptosis of the cells [8]. An increase in the degree of fatty acid saturation and hence, a reduction of the membrane fluidity and permeability of *Dunaliella* sp. isolated from an Antarctic hypersaline lake was observed [51]. Intra-cellular Na^+^ in *D. salina* remains unchanged up to 2.0 M NaCl (117 g L^−1^); thereafter, a significant increase was observed [7]. Glycine betaine and glycerol contents increase concomitantly with salt concentration. Calcium acts as a second messenger in the osmoregulation system of this halotolerant species [6]. Other species as *Chlamydomonas* sp. have depigmented cells following lipid peroxidation of the plasma membrane in the presence of 165 g L^−1^ NaCl [52]. In cyanobacteria, the synthesis of osmolytes depends on their ability to tolerate salt [53]: species with low salt tolerance (up to 0.7 M NaCl) accumulate sucrose and trehalose, species such as *Synechocystis* sp. PCC 6803 with moderate salt tolerance (up to 1.8 M NaCl) accumulates glycosylglycerol [54], and species that tolerate high salt concentration (up to 2.7 M NaCl), such as *Synechococcus* sp. PCC 7418 and *Aphanothece halophytica* [55] accumulate glycine betaine or betaine-glutamate. The frustule of *Cyclotella meneghiniana* contained less silica when cells were exposed to increasing salinity from 4–18 mg L^−1^ [56]. Such a demineralization process could contribute to the NaCl sensitivity of *C. closterium* from the Sfax solar saltern.

On the other hand, carotenoid synthesis is stimulated in adapted cells against high irradiation or/and salinity [37,57]. These pigments dissipate excess light energy via the xanthophyll cycle and act as filters that protect photosynthetic apparatus from photo-oxidation [37]. Moreover, they have antioxidant properties that avoid lipid peroxidation in the photosynthetic apparatus by scavenging singlet oxygen [58,59]. Our results show that high irradiance and high salinity stimulated carotenoids synthesis, especially in *D. salina* (14.82 ± 1.46 µg 10^−6^ cells) and in *P. versicolor* only in the presence of NaCl 80 g L^−1^ (Table 2). These results are consistent with those of other authors [7,50,60]. An enhanced carotenoid production in *Nostoc muscorum* and *Phormidium faveolarum* when light and salinity increased, whereas Chl *a* and phycocyanin content are significantly affected [61].

In microalgae like *Chlamydomonas reinhardtii* and *Dunaliella tertiolecta*, the light-harvesting antenna size is adjusted according to light and salinity [62]. Our results showed that the number of photosystems increased significantly in *D. salina* when light level and salinity increased, while their size remained unchanged. On the contrary, Chl *a* and Chl *c* contents tended to decrease in *C. closterium* leading to a decrease in photosynthesis rate and a lower growth under salt stress. The photosynthetic apparatus adjusts not only the number of photosystems but also its activity according to the light level. Melis [11] showed that the photosynthetic apparatus in *D. salina* was stimulated by high irradiance (2000 µmol photons m^−2^ s^−1^). We only observed this trend between E300 and E500. On a Chl a basis, the net photosynthesis rate in *D. salina* was about twofold that of *P. versicolor* and *C. closterium*. Antenna truncation in the cyanobacterium *Synechocystis* sp. strain PCC6803 results in decreased productivity [63]. The photosynthetic activity also depends on salt concentration. It appears that the photosynthetic apparatus of *D. salina* and *P. versicolor* is better protected against salt stress than in *C. closterium*. NaCl increasing from 0.5–1 M (from 29–58 g L^−1^) leads to the decrease of photosynthesis in *Spirulina platensis* under different light levels (80, 100, 200, and 3500 µmol photons m^−2^ s^−1^) [64,65]. This decrease is a regulation of the photosynthetic activity rather than real damage [64]. Berry et al. [66] suggested that *Spirulina platensis* adapts itself under high salinity by different mechanisms in thylakoid and cytoplasmic membranes, like the regulation of intracellular Na^+^ concentration via a Na^+^-ATPase, ATP being generated by respiration and the cyclic electron transport around PSI [67]. Na^+^-ATPases belonging to the family of P-type ATPases have also been found in marine microalgae, *Tetraselmis viridis* [68], *Heterosigma akashiwo* [69], and *D. maritima* [70]. Adaptation of *Synechocystis* to light and salt stress can be associated with the balance between the rate at which damage was induced and the rate of repair of PSII [5]. To estimate the state of the photosystems of *D. salina*, *C. closterium*, and *P. versicolor*, especially PSII, the fluorescence of Chl a was measured with a modulated fluorometer.

The ratio Fv/Fm has been widely used to assess the extent of photo-inhibition in microalgae [71]. A decrease of Fv/Fm can both be an indicator of PSII damage or a regulation index of electron transport at the PSII level, which leads to heat dissipation of excess light energy. Fv/Fm was almost constant (about 0.7) in both microalgae, but it was lower (about 0.55) in *P. versicolor* [72]. This ratio was defined as an index of the maximum photochemical efficiency of PSII [73], which depends on both F0 and Fv. In cyanobacteria, phycobiliprotein fluorescence interferes with chlorophyll fluorescence which leads to an increase in F0 value. As a consequence, Fv/Fm value decreases [74]. Moreover, the saturating flash detaches phycobiliproteins from the photosynthetic apparatus causing a fluorescence decrease [75]. This reaction is considered a photo-protective mechanism that protects photosynthetic apparatus against high light levels in cyanobacteria. Aquaporins in the cytoplasmic membrane of *Synechocystis* sp. PCC6803 might be necessary for the repair of PSII and PSI photodamage [76].

When photochemistry works, the effective quantum yield (ΦPSII) decreases since a part of PSII centers is reduced (or closed). Under salt stress, the reduction of PSII activity in *D. maritima* leads to an immediate reduction of ΦPSII values [77]. Under our experimental conditions, a decrease of ΦPSII in *D. salina* was measured when irradiance increased and in *C. closterium* when it was submitted to a high salinity and a high light level. We can notice that PN and ΦPSII did not always have the same trends in the diatom and the cyanobacterium (for example, *C. closterium* NaCl 80 g L^−1^, E1000). This absence of a positive correlation between these two parameters is due to salt and/or light impacts on the other components of photosynthetic activity. Allakhverdiev et al. [5] showed that *Synechocystis* sp. (PCC 6803) cells exposition to light (E500) or salt stress (NaCl 29 g L^−1^) led to partial inactivation of PSII. Moreover, the combination of these two stresses induced a complete PSII inhibition. We observed a similar phenomenon with *C. closterium*, which was unable to survive in the presence of NaCl 140 g L^−1^ beyond E500. According to Zakhozhii et al. [77], the reduction of PSII activity is due to structural as well as functional disturbances of PSII and the electron transport chain in *D. maritima*. Despite these disruptions, photosynthetic apparatus continued to operate and produce the energy required for physiological and biochemical processes [7]. Bukhov and Carpentier [78] showed that PSI has a crucial role in producing the energy needed for defense mechanisms against stress. Net photosynthesis as ΦPSII decreased in both the microalgae while ΦPSII values increased, and net photosynthesis decreased in response to salinity rising under the three light levels in *P. versicolor*. In this latter species, PSII could be less affected by salinity than carbohydrate synthesis. Liska et al. [79] showed that photosynthetic activity was over twofold (from 96.8–193.6 µM O_2_ mg^−1^ Chl a h^−1^) in cells grown in 3 M NaCl than in 0.5 M NaCl in *D. salina*. According to these authors, this improvement serves the synthesis of organic solutes and osmolytes. So, cyanobacteria like *Aphanothece* sp., *Phormidium*, or *Oscillatoria* sp. piled up glycine betaine or betaine glutamate in the presence of NaCl 156 g L^−1^ [80]. The effect of salt stress on PSII in cyanobacteria could be attributed to a direct interaction between salt and PSII via cellular components still unknown [74]. Zeng and Vonshak [65] observed that ΦPSII in *Spirulina platensis* decreased by 15% after a 25 h exposure to NaCl 29 g L^−1^ under 100 µmol m^−2^ s^−1^, whereas ΦPSII decreased by about 75% under 200 µmol m^−2^ s^−1^ at the same salinity. However, PSII activity regained its original level after an 80 h exposure showing that, after an initial acclimation phase during which photosynthetic activity was inhibited, a new steady state was established with a recovery of the photosynthetic activity. Our results showed that light level had no significant effect on *P. versicolor* PSII activity.

NPQ increase acquaints about the dissipation of excess light energy as heat radiation when cells are subjected to stress [28]. Our results are in accordance with those of other works [22,71,81], which reported that NPQ increases when microalgae are subjected to salt and/or light stress. Under the most stressful condition, NPQ was 24-fold the value measured in *D. salina* under the control condition, 80-fold in *C. closterium*, and 10-fold in *P. versicolor*. In *C. closterium*, the most NaCl-sensitive species, NPQ reached the value of 21 in the presence of NaCl 80 g L^−1^ and E1000. The xanthophyll-dependent NPQ appeared as an efficient photoprotective mechanism in diatoms [26] since the net photosynthesis of *C. closterium* was stimulated under E1000. Thaipratum et al. [82] proposed that NPQ in *D. salina* is a multi-component process, as it was also shown in the diatom *Phaeodactylum tricornutum* [9].

Reactive oxygen species (ROS) generated by abiotic stresses are scavenged by antioxidative molecules and antioxidative enzyme activities in species having physiological mechanisms to cope with ROS [83]. CAT, SOD, and APX activities were only detected when the three species were cultivated under E1000, except the APX activity in the cyanobacterium *P. versicolor* similar to *Nostoc flagelliforme* [84] and *Cyanobium bacillare* [85], which are also devoid of APX activity. The salinity increase stimulated ROS production and the three enzyme activities studied. Similar results were obtained in *Ulva fasciata* after a 12 h exposure to NaCl 90 g L^−1^ since CAT, Fe-SOD, Mn-SOD, and APX activities were stimulated [86]. Bamary and Einali [87] indicate the induction of oxidative stress in *D. salina* cells reared under 3 M NaCl (175 g L^−1^) by the induction of H_2_O_2_ level.

Rijstenbil [88] showed that salt stress (60 g L^−1^) stimulates the production of ROS in *C. closterium* regardless of light irradiance since SOD and APX activities attained 400 and 35 enzyme units per mg protein, respectively. These values are clearly higher than those obtained in the strain isolated from the Sfax saltern under the most stressful condition (NaCl 140 g L^−1^ and E1000). It is probable that strains living in salt marshes have acquired adaptative mechanisms to salt that are more efficient than in marine strains. Among the salt-adaptative mechanisms, species living in salterns can have a non-enzymatic antioxidative system, a particularly active and/or efficient NaCl exclusion system, and/or an efficient photoprotective system. We also noticed enhanced SOD and CAT activities in *P. versicolor* when the salinity increased, whereas antioxidative enzyme activities in *D. salina* varied slightly when the salinity increased. In this latter species, carotenoid accumulation could play a major role in the antioxidative defense. The increase in SOD, CAT, and APX activities in relation to salt concentration was higher in *C. closterium* than in the two other species. This biochemical response could be related to growth inhibition as in *Chlamydomonas reinhardtii* and *Peridinium gatunense*, in which the highest antioxidative activity precedes cell death, probably due to high antioxidative activity or a metabolite generated by stress that triggers cell death cascade [89].

Despite the stimulation of antioxidative enzyme activities in *C. closterium*, this diatom was more affected by NaCl 140 g L^−1^ than *D. salina* and *P. versicolor*. Salt and high irradiance triggered protective mechanisms that were more efficient in *D. salina* and *P. versicolor* than in *C. closterium*. The maintenance of photosynthetic activity allowed the production of the energy required for physiological and biochemical processes necessary for cell survival (e.g., osmolytes and carotenoids synthesis, antioxidative enzyme activities). In *C. closterium*, antioxidative enzyme activities were triggered, but this defense mechanism was insufficient to cope with NaCl and light stress.

## Figures and Tables

**Table 1:** 18S, 16S rDNA, rbcl, and 5.8S oligonucleotide primers utilized in the present study. Ph: *Phormidium*, NL: *Nitzschia longissima*, DS: *Dunaliella salina*, Nit: *Nitzschia*, cyl: *Cylindrotheca closterium*. ^a^ F: forward primer; R: reverse primer, N: Nested primer.

Primer ^a^	Sequence (5’-3’)	Temperature of Hybridation (°C)
Fph16S	GCAAGCGTTATCCGGAATKAT	61
Rph16S	CCTGTGTTCGCGCTCCCGAA	63
NFph16S	TTATCCGGAATKATTGGGCGT	61
NRph16S	GTTCGCGCTCCCGAAGGCAC	62
F2NL18S	GCGCACCAAGGTAATGATTAA	64
R2NL18S	TTAATCATTACCTTGGTGCGC	60
18SF	CCGGCGATGGATCATTCAAGT	58
18SR	TTCACCGGACCATTCAATCGG	58
rbcL1	AAGGAGAAATHAATGTCT	52
rbcL7	AARCAACCTTGTGTAAGTCTC	52
ITS1	TCCGTAGGTGAACCTGCGG	52
ITS2	TCCTCCGCTTATTGATATGC	52
FDSITS2	AGGCTAGCTCAAGGACCCGT	64
RDSITS2	AGGGCCGAGCCCATGGTCC	66
FNitITS2	TACAACTTTCAGCGGTGGAT	58
RNitITS2	TACCAGAGATAGGACGAGGA	56
FcylITS2	TAACAAGGTTCCGTAGTGAA	56
RcylITS2	TAGCACAAAGGCTACTCTCA	58

**Table 2:** Maximum growth rate, maximum cell density (or Chl a content), pigment contents, and light-harvesting antenna size in *Dunaliella salina*, *Cylindrotheca closterium*, and *Phormidium versicolor* grown in artificial seawater containing NaCl 40, 80, and 140 g L^−1^ under an irradiance of 300, 500, and 1000 µmol photons m^−2^ s^−1^. Means ± SD (*n* = 3), *p* < 0.01 or *p* < 0.001 (depending on the species and the parameter).

Parameters	*Dunaliella salina*								
Irradiance (μmol Photons m^−2^ s^−1^)	300			500			1000		
NaCl (g L^−1^)	40	80	140	40	80	140	40	80	140
Maximum growth rate (day ^−1^)	0.53 ± 0.15 ^aB^	0.61 ± 0.15 ^aC^	0.57 ± 0.23 ^bG^	1.06 ± 0.08 ^cA^	0.95 ± 0.01 ^cD^	0.36 ± 0.05 ^dF^	0.91 ± 0.04 ^eA^	0.80 ± 0.01 ^eE^	0.27 ± 0.06 ^fF^
Maximum cell density (10^6^ cells mL^−1^)	2.02 ± 0.15 ^aA^	2.00 ± 0.12 ^bD^	1.61 ± 0.10 ^cF^	2.89 ± 0.16 ^dB^	2.57 ± 0.02 ^eE^	1.13 ± 0.07 ^fG^	2.37 ± 0.05 ^gC^	2.09 ± 0.01 ^hD^	0.95 ± 0.01 ^iH^
Chl *a* (µg mL−^1^)	2.55 ± 0.17 ^aA^	1.93 ± 0.18 ^bB^	1.62 ± 0.15 ^bE^	3.46 ± 0.31 ^cA^	2.92 ± 0.15 ^dB^	1.67 ± 0.13 ^eD^	2.41 ± 0.32 ^fA^	3.10 ± 0.38 ^fC^	1.56 ± 0.09 ^gD^
Chl *b* (µg mL−^1^)	0.60 ± 0.14 ^aA^	0.50 ± 0.10 ^aC^	0.48 ± 0.05 ^aE^	0.54 ± 0.08 ^bB^	0.56 ± 0.18 ^bC^	0.32 ± 0.05 ^bD^	0.38 ± 0.04 ^cdB^	0.58 ± 0.12 ^cC^	0.35 ± 0.09 ^dD^
Carotenoids (µg mL−^1^)	1.56 ± 0.74 ^aA^	2.57 ± 0.27 ^aC^	4.65 ± 0.45 ^bE^	3.35 ± 0.63 ^cAB^	4.01 ± 1.65 ^cC^	7.94 ± 1.21 ^dF^	4.35 ± 1.07 ^eB^	7.36 ± 1.97 ^eD^	14.82 ± 1.46 ^fG^
Antenna size (Chl *a*/ Chl *b*)	4.4 ± 0.93 ^aA^	5.31 ± 0.95 ^aB^	6.29 ± 1.09 ^aC^	4.90 ± 0.68 ^bA^	5.57 ± 1.96 ^bB^	4.95 ± 0.58 ^bC^	4.10 ± 0.79 ^cA^	3.53 ± 1.06 ^cB^	4.65 ± 1.32 ^cC^
	*Cylindrotheca closterium*								
Maximum growth rate (day^−1^)	0.46 ± 0.07 ^aA^	0.22 ± 0.05 ^aB^	0.04 ± 0.02 ^bE^	0.40 ± 0.05 ^cA^	0.35 ± 0.02 ^cC^	0.00 ± 0.00 ^dD^	0.22 ± 0.12 ^eA^	0.22 ± 0.02 ^eBC^	0.0 ± 0.0 ^fD^
Maximum cell density (10^6^ cells mL^−1^)	0.42 ± 0.04 ^aA^	0.30 ± 0.02 ^bC^	0.22 ± 0.02 ^cG^	0.53 ± 0.07 ^dA^	0.49 ± 0.05 ^eD^	0.06 ± 0.01 ^fF^	0.72 ± 0.06 ^aB^	0.62± 0.05 ^bE^	0.06 ± 0.01 ^cF^
Chl *a* (µg mL−^1^)	0.97 ± 0.20 ^aA^	0.86 ± 0.08 ^aC^	0.56 ± 0.04 ^bE^	0.95 ± 0.02 ^cAB^	0.86 ± 0.08 ^cD^	0 ± 0 ^dF^	1.13 ± 0.09 ^eB^	0.89 ± 0.07 ^eD^	0 ± 0 ^fF^
Chl *c* (µg mL−^1^)	0.40 ± 0.05 ^aA^	0.26 ± 0.06 ^aC^	0.25 ± 0.06 ^bE^	0.21 ± 0.02 ^cB^	0.17 ± 0.05 ^cD^	0 ± 0 ^dF^	0.27 ± 0.05 ^eB^	0.24 ± 0.03 ^eD^	0 ± 0 ^fF^
Fucoxanthin (µg mL−^1^)	0.51 ± 0.02 ^aA^	0.51 ± 0.02 ^aB^	0.30 ± 0.01 ^bD^	0.47 ± 0.13 ^cA^	0.42 ± 0.02 ^cC^	0 ± 0 ^dE^	0.45 ± 0.16 ^eA^	0.51 ± 0.14 ^eC^	0 ± 0 ^fE^
Antenna size (Chl *a*/ Chl *c*)	2.38 ± 0.19 ^aA^	3.39 ± 0.61 ^aC^	2.29 ± 0.55 ^bD^	4.63 ± 0.36 ^cB^	5.33 ± 1.22 ^cC^	0 ± 0 ^a2 dE^	4.20 ± 0.58 ^eB^	3.78 ± 0.16 ^eC^	0 ± 0 ^fE^
	*Phormidium versicolor*								
Maximum growth rate (day^−1^)	0.61 ± 0.07 ^aA^	0.66 ± 0.04 ^aC^	0.29 ± 0.01 ^aD^	0.45 ± 0.04 ^bA^	0.27 ±0.02 ^bB^	0.31 ±0.03 ^bD^	0.35 ± 0.06 ^cA^	0.32 ± 0.04 ^cB^	0.27 ± 0.03 ^cD^
Chl *a* (µg mL^−1^)	1.55 ± 0.23 ^aA^	0.77 ± 0.23 ^aB^	0.71 ± 0.16 ^bD^	1.38 ± 0.49 ^cA^	1.31 ± 0.41 ^cB^	0.17± 0.06 ^dE^	0.94 ± 0.26 ^eA^	2.12 ± 0.03 ^eC^	0.46 ± 0.10 ^fD^
Carotenoids (µg mL^−1^)	0.13 ± 0.01 ^aA^	0.19 ± 0.01 ^bCD^	0.30 ± 0.01 ^bF^	0.25 ± 0.01 ^cB^	0.41 ± 0.03 ^dC^	0.21 ± 0.13 ^dE^	0.14 ± 0.05 ^eA^	0.56 ± 0.08 ^fD^	0.34 ± 0.01 ^fE^

^a–i^: subsets of NaCl levels generated by the TUKEY test under the same light intensity, different numbers indicate a significant difference at 95% level. ^A–H^: subsets of light levels generated by the TUKEY test at the same salinity, different letters indicate a significant difference at 95% level.

**Table 3:** Effect of NaCl and irradiance on net photosynthesis (P_N_), maximum quantum yield (*F_v_*/*F_m_*), the effective quantum yield of PSII (*Φ_PSII_*) and non-photochemical quenching (*NPQ*) in *Dunaliella salina*, *Cylindrotheca Closterium*, and *Phormidium versicolor* grown in artificial seawater containing NaCl 40, 80, and 140 g L^−1^ under 300, 500, and 1000 µmol photons m^−2^ s^−1^. Means ± SE, *p* < 0.001 except for *Φ_PSII_* and net photosynthesis in *Cylindrotheca closterium*: *p* < 0.05 and *p* < 0.01, respectively.

Parameters	*Dunaliella salina*								
Irradiance (μmol Photons m^−2^ s^−1^)	300			500			1000		
NaCl (g L^−1^)	40	80	140	40	80	140	40	80	140
P_N_ (μmol O_2_ h^−1^ mg^−1^ Chl a)	728 ± 35 ^aA^	649 ± 53 ^aD^	467 ± 32 ^bE^	1017 ± 36 ^cB^	711 ± 120 ^dD^	447 ± 27 ^eE^	840 ± 27 ^fC^	668 ± 52 ^gD^	367 ± 28 ^hF^
*F_v_*/*F_m_*	0.71 ± 0.02 ^abA^	0.75 ± 0.01 ^bB^	0.74 ± 0.04 ^aC^	0.75 ± 0.02 ^bA^	0.67 ± 0.12 ^bB^	0.75 ± 0.01 ^bC^	0.78 ± 0.03 ^aA^	0.75 ± 0.01 ^aB^	0.64 ± 0.02 ^bD^
*Φ_PSII_*	0.32 ± 0.09 ^aA^	0.28 ± 0.13 ^aC^	0.37 ± 0.17 ^aD^	0.31 ± 0.02 ^bA^	0.22 ± 0.01 ^cC^	0.23 ± 0.01 ^bcE^	0.23 ± 0.02 ^deB^	0.16 ± 0.01 ^dC^	0.11 ± 0.01 ^eE^
*NPQ*	0.23 ± 0.11 ^aA^	0.23 ± 0.06 ^bC^	0.49 ± 0.19 ^cD^	0.74 ± 0.12 ^dA^	1.01 ± 0.07 ^cC^	0.71 ± 0.12 ^dE^	1.3 ± 0.07 ^fB^	2.48 ± 0.44 ^eC^	5.97 ± 0.39 ^eE^
	*Cylindrotheca closterium*								
P_N_ (μmol O_2_ h^−1^ mg^−1^ Chl *a*)	387 ± 69 ^aA^	343 ± 19 ^abB^	243 ± 32 ^b^	427 ± 39 ^cA^	365 ± 53 ^cB^	nd	425 ± 34 ^dA^	287 ± 71 ^eB^	nd
*F_v_*/*F_m_*	0,71 ± 0.02 ^abA^	0.74 ± 0.09 ^aB^	0.76 ± 0.11 ^b^	0,68 ± 0.04 ^cA^	0,76 ± 0.02 ^cB^	nd	0.75 ± 0.03 ^dA^	0.73 ± 0.04 ^dB^	nd
*Φ_PSII_*	0.51 ± 0.05 ^aA^	0.39 ± 0.13 ^aD^	0.20 ± 0.08 ^ab^	0.25 ± 0.03 ^cB^	0.37 ± 0.02 ^dE^	nd	0.20 ± 0.03 ^eC^	0.30 ± 0.01 ^eF^	nd
*NPQ*	0.51 ± 0.10 ^aA^	0.31± 0.18 ^aC^	8.05 ± 2.24 ^b^	4.18 ± 0.19 ^cAB^	10.89 ± 2.47 ^cB^	nd	7.76 ± 0.15 ^dB^	21.31 ± 4.63 ^dD^	nd
	*Phormidium versicolor*								
P_N_ (μmol O_2_ h^−1^ mg^−1^ Chl*a*)	552 ± 66 ^aA^	432 ± 26 ^aB^	278 ± 42 ^bC^	421 ± 52 ^cA^	370 ± 79 ^cdB^	274 ± 28 ^dC^	474 ± 46 ^eA^	372 ± 33 ^fB^	279 ± 37 ^fC^
*F_v_*/*F_m_*	0.39 ± 0.04 ^aA^	0.46 ± 0.08 ^aCD^	0.50 ± 0.07 ^aE^	0.35 ± 0.05 ^bA^	0.33 ± 0.04 ^bC^	0.45 ± 0.09 ^bE^	0.55 ± 0.07 ^cB^	0.55 ± 0.10 ^cD^	0.50 ± 0.19 ^cE^
*Φ_PSII_*	0.28 ± 0.04 ^aB^	0.26 ± 0.03 ^aC^	0.52 ± 0.03 ^bF^	0.15 ± 0.05 ^cA^	0.16 ± 0.01 ^cD^	0.21 ± 0.05 ^cG^	0.14 ± 0.05 ^eA^	0.38 ± 0.04 ^dE^	0.47 ± 0.09 ^dF^
*NPQ*	0.12 ± 0.03 ^aA^	0.19 ± 0.13 ^aC^	0.21± 0.14 ^aE^	0.14 ± 0.07 ^bAB^	0.15 ± 0.02 ^bC^	0.67 ± 0.24 ^cE^	0.65 ± 0.35 ^dB^	1.5 ± 0.87 ^dB^	0.33 ± 0.21 ^dE^

^a–i^: subsets of NaCl levels generated by the TUKEY test under the same light intensity, different numbers indicate a significant difference at 95% level. ^A–H^: subsets of light levels generated by the TUKEY test at the same salinity, different letters indicate a significant difference at 95% level. nd: not determined.

## Data Availability

Not applicable.
